# A Mixed Methods Study on Character Traits of Nursing Students and Faculty Influencing a 100% Passing Rate in the Nursing Licensure Exam in the Philippines

**DOI:** 10.7759/cureus.69911

**Published:** 2024-09-22

**Authors:** Rudena A Madayag, Maria Fe M Mallari, Doroteo S Dizon, Rei Angelo P Mangibin, Jasleen S Yumang, Jonel D Mallari, Ma. Corazon M Tanhueco, Diane Anne A Lozano, Zenaida S Fernandez

**Affiliations:** 1 College of Nursing, Graduate School, Angeles University Foundation, Angeles, PHL; 2 College of Nursing, Angeles University Foundation, Angeles, PHL

**Keywords:** academic achievement, character strengths, faculty influence, nurse licensure examination, nursing education and practice

## Abstract

Introduction: This study explores the character traits contributing to the 100% passing rate of nursing graduates in the Nursing Licensure Examination (NLE) in the Philippines over the past decade. Understanding these traits provides insights into the factors driving academic and professional success in nursing.

Methods: Employing a convergent parallel mixed methods design, this study combined quantitative and qualitative approaches. Data were collected through electronic surveys from 222 nursing alumni and 39 faculty members, focusing on character traits and behaviors linked to NLE success. The quantitative data were analyzed using Statistical Product and Service Solutions (SPSS, version 29; IBM SPSS Statistics for Windows, Armonk, NY) for descriptive statistics and factor analysis. Additionally, in-depth interviews with 21 alumni and 15 faculty members provided qualitative insights. Data saturation was reached with 11 faculty and 13 alumni, ensuring comprehensive coverage of the participants' experiences.

Results: Quantitative results from factor analysis showed that spirituality, love of learning, honesty, and kindness are among the qualities that are necessary for success in NLE. These character traits have a significant impact on both career readiness and academic performance. Qualitative results revealed the importance of resilience, faculty support, and peer relationships in fostering these attributes. It became evident that building a healthy learning environment required the support of peers and professors and that resilience was necessary to manage the stress of tests. Both datasets emphasized the need for a strong character and academic achievement in conjunction with long-term success.

Discussion: The study highlights that obtaining a high NLE passing rate is mostly dependent on academic knowledge in addition to character attributes. Character education techniques such as role-playing, reflective writing, and service-learning can be incorporated into nursing curricula to improve attributes such as empathy and resilience. Continuous faculty development and structured mentorship are also crucial.

Conclusion: Character development must be incorporated into academic learning in nursing education. Focusing on qualities such as empathy, resilience, and ethical judgment, along with implementing character education tactics such as service-learning and reflective writing, are crucial to prepare graduates to succeed in professional practice and board exams. A comprehensive approach that blends character development with academic achievement produces skilled and compassionate healthcare professionals.

## Introduction

In the realm of nursing education, the Nursing Licensure Examination (NLE) is a pivotal moment for aspiring healthcare professionals. It serves as the gateway to practicing nursing, determining the future trajectory of being a registered nurse. It is well known that successful candidates in nurse licensure examinations entail a standard curriculum that covers the program learning outcomes and their indicators aligned with the competencies in nursing practice standards, as well as the provision of an excellent learning environment with well-equipped laboratory school facilities. Having supportive administrators and educators in the academe are essential components, together with the consistent review classes, that were initially identified as contributory factors in the excellent nursing board performance rating.

The National Council of State Boards of Nursing pointed out that quality patient care emanates from a professional nurse who employs competent and safe nursing practices [1]. Ensuring competent and safe nursing practice involves the establishment of requirements, rules, practice regulations, and laws by nurse regulatory boards [2]. A nurse's professional license is obtained after passing a nurse licensure examination, which bestows upon the nurse the right to practice the profession and guarantees entry-level aptitudes for safe nursing practice. Many countries administer their licensure exams for nurses, and the Philippine Nursing Licensure Exam (PNLE) in the Philippines is among the national exams certain countries require before nurse licensure.

Studies from other countries have sought to determine the predictors of a successful nurse licensure examination [3]. For example, in the United States, grade point average (GPA) and participation in comprehensive mock exams were identified as predictors of success on the National Council Licensure Examination - Registered Nurses (NCLEX-RN). Additionally, nurse graduates with high scores on the Health Education Systems Incorporated Exit Exam who engaged in stress management activities and proactive test preparation were found to be successful on their nurse licensure exams [4]. In the Philippines, factors such as the student-faculty ratio and other nursing school characteristics, including location, size, type, and establishment year, were linked with nurse licensure exam passing rates [5]. Other research has found that terminal competency assessments, GPA, intelligence quotient, college admission tests, and pre-board examinations were significantly associated with nurse licensure exam performance [6]. Additional studies identified GPA, school performance, time management, test-taking skills, and group studies as indicators of success on nurse licensure examinations [7].

It has been highlighted that nurse educators play an essential role in achieving nursing students’ learning outcomes [8]. Therefore, nurse educators should possess core competencies to assist students throughout the educational process. Teaching quality comprises knowledge, competencies, and values [9]. Character traits of educators significantly impact successful learning outcomes, including students passing licensure examinations. Differences in the quality of nursing education programs across various domains have been noted [10].

Character strengths are known to predict educational outcomes beyond general personality traits and cognitive ability [11], but their specific role in promoting success and positive learning experiences varies across educational settings, especially when it comes to rigorous tests such as the NLE. In nursing education, where knowledge and skills in providing quality care for patients are crucial, the development of professional and character attributes holds equal importance. Given that nurses are at the frontline of healthcare, personality growth is particularly relevant. The combination of academic success and personal values can profoundly influence long-term professional competence, ethical behavior, and resilience in the workplace [12].

Furthermore, there is a gap in the literature concerning the various ways in which certain character traits, specifically integrity, compassion, and resilience, can contribute to passing licensure exams and thriving in the demanding nursing profession. Existing studies often emphasize academic performance indicators, such as GPA and thorough board exam reviews [13], while overlooking how character development during the educational process influences licensure success. This study seeks to bridge that gap by probing into the unique personal traits associated with exceptional performance in nursing board exams and exploring how character development can be integrated into nursing education programs to enhance both academic outcomes and real-world readiness. By addressing this gap, the research aims to contribute valuable insights into how character traits play a pivotal role in the success of future registered nurses.

Specifically, this study addresses the following research questions: What character attributes, both of nursing students and faculty members, contribute to a 100% passing rate in the nursing board exams? How do nursing students' and faculty members' attitudes, values, and behaviors impact board exam success? What strategies or practices can be implemented in nursing education programs to enhance character development and ultimately improve board exam performance? Understanding these factors will provide valuable insights for improving nursing education programs globally.

## Materials and methods

The primary objectives of this study were to identify character traits linked to a 100% passing rate in the PNLE and to understand how these traits contributed to both academic and professional success. A convergent parallel mixed methods approach was employed to extensively look into the role of character attributes in achieving a 100% passing rate in the PNLE, providing a comprehensive understanding of the complex nature of these character traits and their impact on exam success over the past decade.

Considering it was following the study's goal, the study was carried out at a university in Pampanga, Philippines, which had continuously maintained a 100% passing rate for the last 10 years.

Quantitative component

The quantitative component employed the Values in Action (VIA) Classification of Character Strengths and Virtues questionnaire, a free online survey that is widely utilized for assessing character strengths. This validated and reliable tool was chosen for its broad application in research and its accessibility for evaluating character traits among nursing students and faculty members separately. The questionnaire assesses 24 specific character strengths, which are detailed and accessible through the online assessment survey [14]. The inclusion of these 24 character strengths in the questionnaire allows for a nuanced analysis of traits influencing exam success.

While the VIA survey is a widely used, free online tool, its application in the Filipino cultural and educational context is of particular interest. The survey is designed to be universally applicable, and no formal adaptation was required for this study. However, we recognized that cultural differences might influence the expression and interpretation of character traits. To address this, we incorporated qualitative insights to capture any culturally specific nuances related to character traits, thereby enhancing the relevance of the VIA tool in the Filipino setting.

The reliability of the results was reinforced by the consistency of character trait rankings for alumni spanning several years and for faculty participants across various ages, as well as by high loading patterns in factor analysis. The reliability of the results is further strengthened by descriptive statistics reflecting variability in trait perceptions.

Qualitative component

For the qualitative component, in-depth interviews were conducted via videoconferencing with 21 alumni and through one-on-one interviews with 15 faculty members.

The study aimed to explore both alumni and faculty perspectives on the critical factors influencing success in the NLE. For alumni, the central question addressed was the following: What character traits and faculty influences were crucial for your success in the NLE, and how did they impact your preparation and performance? To delve deeper, alumni were asked to provide specific examples of how resilience and determination shaped their exam preparation and overall performance. They were also queried about the role of faculty support and role modeling in their preparation and performance, as well as the ways in which personal growth contributed to their success in the licensure exams.

For faculty, the main question sought to uncover was the following: What do you believe are the key character traits and instructional strategies that contribute to nursing students' success in the NLE, and how do you implement these in your teaching? Follow-up inquiries aimed to gather detailed examples of how faculty has cultivated resilience and determination among students. Faculty were also asked to describe how they tailor their support and mentorship to enhance students' preparation for the board exams and to reflect on how their own character traits and teaching approaches influence students’ success in the licensure exams.

Participants were selected based on their significant contributions to the institution's success. During the interviews, participants were guided through the process and allowed to use their preferred language to ensure open and honest feedback. Certificates of participation and small tokens of appreciation were provided as a gesture of gratitude. Data saturation was reached with 11 faculty members and 13 alumni, indicating that no new information emerged beyond this point. This saturation level confirmed that the qualitative data were comprehensive and reflective of the participants' experiences and insights.

Capturing multiple perspectives from relevant experiences and expertise ensured the reliability of the findings. Responses from both alumni and faculty were cross-referenced for triangulation purposes. Member checking was used to validate transcripts, ensuring accuracy and minimizing bias. The consistent use of interview guides and the attainment of data saturation supported the robustness of the qualitative findings, demonstrating that the sample size was sufficient to represent the primary themes of the study.

Data sampling and collection

Participants were purposively selected from a higher education institution in Pampanga, Philippines, known for its consistent 100% passing rate in the PNLE over the past 10 years. For the quantitative component, data were collected using electronic surveys from a total of 222 nursing alumni and 39 faculty members. The survey focused on character attributes, attitudes, values, and behaviors contributing to board exam success. To ensure relevance and accuracy, the study included alumni who graduated within the last five years and faculty members with teaching experience of over a year and a postgraduate degree. Individuals who did not meet these criteria or chose not to participate were excluded from the study.

Data analysis

The quantitative data collected from the survey were analyzed using Statistical Product and Service Solutions (SPSS, version 22.0; IBM SPSS Statistics for Windows, Armonk, NY). Descriptive statistics, including means, standard deviations, and frequency distributions, were used to summarize the prevalence and importance of specific character traits associated with board exam success. Factor analysis was conducted to identify and interpret underlying dimensions of character traits, grouping traits into distinct factors based on their loadings.

The qualitative data obtained from interviews were transcribed and analyzed through thematic analysis. Emerging themes and sub-themes were identified, organized, and categorized based on the character traits, faculty influence, curriculum design, and personal development highlighted by participants. This analysis provided a comprehensive understanding of the factors contributing to board exam success and character development among nursing students and faculty members.

## Results

Table 1 presents a comprehensive overview of the demographic characteristics of the nursing alumni participating in the study. The data reveals that a substantial majority of the alumni graduated in 2023 (43.60%), followed by 2022 graduates (27.30%), indicating a strong focus on recent graduates. The representation from earlier years is comparatively lower, with only 0.90% from 2021 and varying percentages from 2020, 2019, and 2018. This distribution reflects the study’s emphasis on contemporary trends and experiences among recent alumni.

**Table 1:** Demographic Characteristics of Nursing Alumni Frequencies (f) and percentages (%) are based on the total number of respondents (n=222) for each demographic category.

Characteristic		Category	Frequency	Percent
	Gender	Male	55	24.8
		Female	167	75.2
		Total	222	100%
	Years Graduated	2023	99	43.60
		2022	61	27.30
		2021	2	0.90
		2020	19	8.4
		2019	17	7.50
		2018	23	10.10
		Total	222	100%
	Age	21	2	0.90
		22	30	13.5
		23	76	34.2
		24	51	23
		25	27	12.2
		26	16	7.2
		27	2	0.90
		30	9	4.1
		32	6	2.7
		35	3	1.4
		Total	222	100%

In terms of gender, the table shows a notable predominance of female alumni, who make up 75.2% of the sample, while male alumni constitute 24.8%. This gender distribution is consistent with broader trends in the nursing profession, where women typically represent a larger proportion of the workforce. The age distribution further highlights that the majority of participants are relatively young, with 48.6% between the ages of 21 and 23, and the largest single age group being 23 years old (34.2%). This suggests a younger demographic that is predominantly composed of recent graduates in the early stages of their careers. The data on age indicate a decrease in participation from older alumni, which may influence the generalizability of findings to more experienced professionals.

To address the potential skewness due to the higher representation of recent graduates, it is important to note that focusing on alumni who recently passed the NLE provides relevant insights into the traits and experiences that contribute to immediate exam success. Their feedback reflects the current standards and challenges of the nursing profession, which can be crucial for improving preparation strategies. Nonetheless, we acknowledge the need for a broader longitudinal perspective, and future research should aim to include a more diverse range of graduation years to capture a comprehensive view of character development over time and its implications for long-term success in the nursing profession.

The factor analysis of character traits among nursing alumni revealed five distinct components, each representing unique clusters of traits (Table 2). This analysis was conducted using principal component analysis (PCA) with Varimax rotation. The Kaiser-Meyer-Olkin (KMO) measure of sampling adequacy was 0.445, which is below the ideal threshold and suggests that some variables might not be ideally suited for factor analysis. Nevertheless, Bartlett's test of sphericity showed a chi-square value of 206.517 with 45 degrees of freedom and a significance level of p < 0.001, justifying the use of factor analysis.

**Table 2:** Factor Analysis of Character Traits for Alumni Factor loadings show the correlation between traits and components: High loadings (absolute values between 0.5 and 1.0) indicate strong relationships, moderate loadings (0.3-0.5) show moderate relationships, and low loadings (below 0.3) reflect weak relationships. Negative loadings suggest an inverse relationship with the component.

Character Trait	Factor 1	Factor 2	Factor 3	Factor 4	Factor 5
Honesty	0.288	0.489	0.122	0.395	-0.550
Kindness	-0.433	0.172	0.557	0.033	0.268
Fairness	0.141	-0.633	-0.144	0.328	0.226
Love	-0.318	0.196	-0.300	0.730	0.238
Hope	0.406	0.283	-0.403	-0.217	0.632
Spirituality	0.265	0.782	0.001	0.044	0.209
Humor	0.302	-0.116	0.747	0.026	0.240
Love of Learning	0.428	-0.048	0.508	0.219	0.148
Judgment	0.552	-0.246	-0.114	0.447	0.001
Teamwork	0.703	-0.064	-0.122	-0.263	-0.186

Factor 1 shows high positive loadings for Teamwork (0.703) and Love of Learning (0.428), along with a moderate loading for Honesty (0.288). This suggests that the component represents traits related to collaboration, intellectual curiosity, and ethical behavior, with an emphasis on cooperative and collective efforts. Factor 2 has a high positive loading for Spirituality (0.782) and moderate loadings for Kindness (0.172) and Fairness (-0.633). This reflects traits associated with empathy, compassion, and ethical considerations, with Fairness having a negative loading that suggests an inverse relationship with other traits in this component, highlighting a dimension of spiritual and compassionate qualities. Factor 3 features high loadings for Humour (0.747) and Love of Learning (0.508), indicating an association with a sense of humor and a passion for learning. This suggests that individuals who score high on this component are likely to possess an open-minded and enjoyable approach to both learning and interactions. Factor 4 has a high positive loading for Love (0.730) and a moderate negative loading for Hope (-0.217), representing traits related to optimism and affection, with a nuanced relationship indicated by the negative loading for Hope, focusing on positive emotions and personal connections. Finally, factor 5 demonstrates a high positive loading for Hope (0.632) and a moderate negative loading for Teamwork (-0.186). This component is associated with traits related to resilience and a positive outlook, with the negative loading for Teamwork highlighting a distinct aspect where hope is emphasized more prominently.

Overall, the factor analysis results suggest that the character traits assessed among nursing alumni can be grouped into five distinct dimensions, each representing a unique combination of related traits. These findings provide valuable insights into the underlying factors that may influence the development and expression of these character traits in nursing professionals. The specific factor values (e.g., 0.288, 0.489, etc.) indicate the strength of the relationship between each character trait and the corresponding factor. Higher absolute values suggest a stronger association.

The table demonstrates that Honesty remains the most consistently valued trait among nursing alumni across multiple years, highlighting its enduring importance in the profession (Table 3). Alongside Kindness, which also frequently appears at the top of the rankings, these traits reflect a strong emphasis on integrity and compassion. The stability of these top traits underscores their central role in successful nursing practice and professional interactions over time.

**Table 3:** Character Traits Ranked by Year Graduated Top 10 character traits ranked by frequency among nursing alumni who graduated between 2018 and 2023. The values in parentheses indicate the number of alumni who exhibited each trait in the respective year.

RANK	2023 (n=99)	f	2022 (n=62)	f	2021 (n=2)	f	2020 (n=19)	f	2019 (n=17)	f	2018 (n=23)	f
1	Honesty	26	Honesty	19	Fairness	1	Honesty	7	Honesty	5	Honesty, Love, & love of learning	4
2	Kindness	19	Kindness	13	Kindness & leadership	1	Kindness & fairness	5	Kindness	4	Kindness	4
3	Fairness	12	Love	9	Appreciation of beauty and excellence & spirituality	1	Gratitude & love	3	Fairness	4	Fairness	4
4	Love	14	Humor	8	Teamwork & fairness	1	Humor & gratitude	4	Humor, spirituality, perseverance & fairness	2	Hope	4
5	Hope, spirituality, gratitude	11	Spirituality	10	Perseverance & gratitude	1	Spirituality	4	Hope	5	Appreciation of beauty and excellence	3
6	Judgment	15	Gratitude	15	Hope	1	Creativity	3	Honesty	3	Judgment	4
7	Love of learning	13	Fairness & perseverance	8	Humility & leadership	1	Love of learning	3	Love of learning, kindness & teamwork	3	Love of learning	4
8	Fairness	11	Fairness	8	Honesty & spirituality	1	Fairness	3	Love	3	Gratitude	3
9	Perseverance	11	Perseverance	9	Fairness & teamwork	1	Perseverance	3	Hope, spirituality & fairness	2	Fairness	3
10	Hope	11	Hope & judgment	8	Perseverance & love of learning	1	Hope	5	Perseverance, judgment & Appreciation of beauty and excellence	3	Perseverance	6

Over the years, there has been a noticeable shift towards greater emphasis on traits such as Fairness, Love, and Gratitude. For instance, Fairness and Love have gained prominence in recent years, indicating an increasing recognition of the importance of equitable and empathetic behavior. The rise in the importance of traits such as Perseverance, Hope, and Spirituality reflects evolving professional challenges and a growing appreciation for emotional resilience and optimism in the nursing field. This evolution in valued traits highlights the dynamic nature of professional values as alumni adapt to changing contexts and demands (Table 4).

**Table 4:** Summary of Top 10 Character Traits Among Nursing Alumni The mean, median, mode, standard deviation, variance, minimum, maximum, and range are calculated based on the frequency of each trait across the different years. Higher values indicate greater prevalence and consistency of the trait among the alumni.

Character Trait		Mean	Median	Mode	Standard Deviation	Variance	Minimum	Maximum	Range
	Honesty	25.17	25.5	26	8.32	69.22	4	26	22
	Spirituality	24.17	24.5	11	7.98	63.68	0	29	29
	Hope	21.67	21.5	11	7.09	50.26	0	26	26
	Humor	20.83	20.5	9	6.77	45.83	0	25	25
	Love	20.17	20	14	6.32	39.94	0	21	21
	Teamwork	16.33	16	5	5.46	29.83	0	18	18
	Kindness	15.17	15	10	5.76	33.23	2	19	17
	Love of learning	15.67	15.5	9	5.76	33.23	0	16	16
	Fairness	12.5	12	7	3.94	15.54	0	15	15
	Curiosity	13.67	13.5	8	4.24	18.02	0	13	13

The analysis of character trait profiles among nursing alumni revealed a consistent pattern of core values. Honesty, spirituality, love, and hope emerged as the top traits, demonstrating their enduring importance in the nursing profession. While these core traits were frequently reported, traits such as curiosity, perspective, and zest exhibited more variability, suggesting their prominence might be influenced by individual differences or external factors.

Specifically, honesty and spirituality consistently ranked high in terms of mean, median, and mode. For example, honesty had a mean of 25.17, a median of 25.5, and a mode of 26, indicating that it was frequently reported and consistently present among alumni. Similarly, spirituality had a mean of 24.17, a median of 24.5, and a mode of 11, showcasing its high prevalence and consistency.

In contrast, traits such as curiosity, perspective, and zest demonstrated lower mean and median values, suggesting less consistency in their reporting. For instance, curiosity had a mean of 13.67, a median of 13.5, and a mode of 8, indicating a wider range of responses and less consistent presence among alumni.

These findings highlight the importance of cultivating a diverse range of character traits in nursing students, beyond the traditional values of honesty, kindness, and fairness. By fostering these qualities, nursing programs can contribute to the development of well-rounded and compassionate professionals who are equipped to meet the evolving needs of patients and healthcare systems.

The demographic profile of the faculty members revealed a well-balanced composition in terms of gender and position, with a majority of female representation and a mix of clinical instructors and professors (Table 5). The faculty members demonstrated a strong educational foundation, with most holding Master's degrees and a significant number pursuing or having completed doctoral studies. In terms of experience, the faculty exhibited a diverse range of service years, ensuring a balance of experienced and newer faculty members. The relatively young average age suggested a dynamic and evolving faculty profile. These demographic characteristics could potentially influence the teaching styles, experiences, and perspectives of the faculty members, which may in turn impact the nursing students they teach.

**Table 5:** Demographics of Faculty Staff Frequencies and percentages are based on the total number of faculty members surveyed (n = 39).

Characteristic		Category	Frequency	Percent
	Gender	Male	15	38.5
		Female	24	61.5
		Total	39	100
	Position	Clinical Instructor	20	51.3
		Professor (Assistant, Associate & Professor)	19	48.7
		Total	39	100%
	Highest Educational Attainment	RN	5	12.8
		RN, MAN, MN	20	51.3
		PhD candidate	12	30.8
		PhD	2	5.1
		Total	39	100%
	Years of service	2-5 years	15	38.5
		5-10 years	7	17.9
		10-15 years	5	12.8
		15-20 years	7	17.9
		>20 years	5	12.8
		Total	39	100%
	Age	33.00	2	5.1
		34.00	2	5.1
		35.00	3	7.7
		37.00	1	2.6
		38.00	2	5.1
		39.00	1	2.6
		40.00	4	10.3
		43.00	2	5.1
		44.00	1	2.6
		45.00	2	5.1
		47.00	2	5.1
		48.00	1	2.6
		49.00	1	2.6
		50.00	3	7.7
		51.00	3	7.7
		52.00	2	5.1
		54.00	1	2.6
		56.00	2	5.1
		57.00	1	2.6
		58.00	1	2.6
		59.00	1	2.6
		60.00	1	2.6
		Total	39	100%

The table analysis (Table 6) reveals that Honesty is consistently the most valued character trait among faculty members across all age groups, with the highest frequency observed in those over 50 years old (f=9). This suggests that honesty is a foundational value that remains vital throughout a faculty member's career. Similarly, Kindness is highly ranked across all age groups, particularly in the 30-35, 40-45, and over 50 age brackets (f=3, f=3, and f=6, respectively), highlighting its universal importance in fostering positive professional relationships.

**Table 6:** Character Traits of Faculty Ranked by Age Group The table presents the top 10 character traits ranked by frequency among nursing alumni categorized into four age groups.

Rank	30-35	f	35-40	f	40-45	f	45-50	f	>50	f
1	Honesty	3	Honesty	4	Honesty	3	Love	2	Honesty	9
2	Kindness	2	Kindness	3	Kindness	3	Spirituality	3	Kindness	6
3	Love	2	Love, humor & creativity	3	Kindness & fairness	2	Kindness & Fairness	2	Love	6
4	Spirituality, perspective & humor	1	Humor	2	Humor	3	Hope	2	Fairness	2
5	Teamwork, spirituality & perseverance	1	Bravery & spirituality	2	Spirituality	2	Teamwork	2	Spirituality	4
6	Leadership, Judgment & creativity	1	Gratitude	2	Creativity, gratitude & fairness	1	Kindness, love, gratitude, creativity & Love of learning	1	Gratitude	5
7	Zest, perseverance, kindness, forgiveness, creativity & Love of learning	1	Love of learning	3	Love of learning	2	Gratitude	2	Love of learning	4
8	Gratitude	2	Zest, curiosity, hope, spirituality, perseverance, love & forgiveness	1	Fairness	2	Zest, prudence, love, forgiveness, & love of learning	1	Gratitude	3
9	Fairness	2	Curiosity & fairness	2	Perseverance	2	Spirituality	2	Fairness	5
10	Perseverance	2	Bravery, appreciation of beauty, perseverance, love, judgment & forgiveness	1	Hope	3	Perseverance	3	Perseverance	4

As faculty members age, traits such as Spirituality and Love gain prominence, particularly in the 35-40, 45-50, and over 50 groups, indicating a shift towards more reflective and interpersonal values with professional maturity. Traits such as Humor, Gratitude, and Fairness are consistently valued, though their frequency varies across age groups. Humor is notably important among those aged 35-40 and 40-45, while Fairness is highly regarded in faculty members over 50 (f=5). Additionally, Love of learning is consistently present, underscoring the importance of continuous education and intellectual growth throughout an academic career. These findings suggest that, while some traits remain consistently important, others may become more valued as faculty members grow older, reflecting evolving priorities and professional development.

A factor analysis was conducted to uncover the underlying dimensions of character traits among faculty members (Table 7). The KMO measure of sampling adequacy was 0.482, which falls short of the recommended threshold of 0.6, suggesting that the sampling adequacy might be suboptimal. However, Bartlett’s test of sphericity was significant, with a chi-square value of 69.467 and a p-value of 0.011, confirming that the correlations among variables were sufficient to proceed with the factor analysis.

**Table 7:** Factor Analysis of Character Traits for Faculty Factor loadings show the correlation between traits and components: High loadings (absolute values between 0.5 and 1.0) indicate strong relationships, moderate loadings (0.3-0.5) show moderate relationships, and low loadings (below 0.3) reflect weak relationships. Negative loadings suggest an inverse relationship with the component.

Component	Character Trait	Factor 1	Factor 2	Factor 3	Factor 4
1	Honesty	0.609	0.603	0.039	-0.110
2	Kindness	0.103	-0.796	-0.112	0.021
3	Fairness	-0.147	0.738	-0.040	-0.085
4	Teamwork	0.002	0.063	-0.706	0.146
5	Hope	-0.138	-0.120	-0.146	0.794
6	Love or learning	0.547	-0.122	0.244	0.575
7	Judgment	0.798	-0.140	0.215	-0.122
8	Spirituality	0.785	-0.135	-0.261	-0.054
9	Humor	0.213	-0.055	0.523	-0.539
10	Gratitude	-0.018	0.182	0.780	0.046

PCA with Varimax rotation was performed, revealing four distinct factors based on eigenvalues greater than 1. The first factor, with an eigenvalue of 3.45, included high loadings for Judgment (0.798), Spirituality (0.785), and Honesty (0.609), reflecting traits related to reflective and ethical behavior. The second factor, with an eigenvalue of 2.30, displayed high negative loadings for Kindness (-0.796) and positive loadings for Fairness (0.738), indicating a dimension focused on empathy and fairness, separate from other traits. The third factor, having an eigenvalue of 1.85, included high loadings for Fairness (-0.706) and Gratitude (0.780), representing a dimension associated with fairness and appreciation. Lastly, the fourth factor, with an eigenvalue of 1.12, encompassed Hope (0.794), Teamwork (0.794), and Love of learning (0.575), reflecting traits related to optimism, collaboration, and a passion for learning.

The rotation converged in six iterations, and the results highlight how various character traits are grouped into distinct components based on their factor loadings. This analysis provides a structured understanding of faculty attributes, illustrating the multifaceted nature of professional qualities among faculty members.

The analysis of the character traits among faculty members reveals notable insights into the perceived importance of various traits (Table 8). The mean rankings show that traits such as Honesty (M=4.92), Kindness (M=8.49), and Love (M=8.95) are considered highly significant, indicating that these qualities are highly valued within the academic context. Gratitude and Fairness also feature prominently with means of 11.31 and 11.72, respectively, suggesting that these traits are seen as essential for fostering a positive and equitable environment. The high mean rankings of these traits emphasize their role in shaping interactions and institutional culture.

**Table 8:** Summary of Top 10 Character Traits Among Faculty Members The mean reflects the average ranking for each character trait, while the median denotes the midpoint of the rankings. The mode represents the most frequently observed ranking, and the standard deviation quantifies the variability of the rankings. The minimum and maximum values indicate the lowest and highest rankings, respectively, with the range representing the difference between these extreme values.

Character Trait		N	Mean	Median	Mode	Standard Deviation	Minimum	Maximum	Range
	Honesty	40	4.92	1.00	1.00	4.96	1.00	14.00	41
	Kindness	40	8.49	9.00	9.00	4.54	1.00	20.00	28
	Love	40	8.95	10.00	10.00	4.45	1.00	17.00	26
	Gratitude	40	11.31	13.00	13.00	5.80	1.00	25.00	26
	Fairness	40	11.72	12.00	12.00	6.57	1.00	24.00	25
	Judgment	40	7.15	4.00	4.00	6.89	1.00	26.00	24
	Forgiveness	40	7.87	5.00	3.00	6.64	1.00	26.00	19
	Creativity	40	9.95	9.00	4.00	6.39	2.00	26.00	19
	Love of learning	40	10.36	9.00	5.00	6.73	1.00	23.00	18
	Spirituality	40	11.87	11.00	11.00	5.36	1.00	24.00	14

The variability in the rankings, as indicated by the standard deviations, reflects differing opinions on the importance of these traits. For instance, Creativity (SD=6.39) and Love of learning (SD=6.73) exhibit notable variability, suggesting a broader range of perceptions about their significance compared to more consistently valued traits such as Honesty (SD=4.96). The range values further highlight the disparity in how these traits are ranked, with Gratitude and Fairness showing a narrower range, indicating a consensus on their importance. This variability underscores the diverse priorities among faculty members, influencing the emphasis placed on various character traits in academic and professional settings.

Table 9 highlights key factors that contribute to nursing alumni's success in the NLE through four central themes. Character traits for success emphasize personal qualities such as resilience, determination, and ethical judgment, which are crucial for overcoming academic challenges and excelling in the nursing profession. Faculty influence and support play a significant role, with faculty members modeling professional behavior, offering empathy, and providing structured guidance that effectively prepares students for their exams.

**Table 9:** Themes and Perspectives on Factors Influencing Nursing Students' Success in Board Examinations: Insights From Alumni and Faculty The table provides a detailed summary of the qualitative insights from nursing alumni and faculty, highlighting the key factors that contribute to students' success in board exams.

Theme		Sub-theme	Alumni Perspectives	Faculty Perspectives
	Character Traits for Success	Resilience and Determination	"Resilience and determination are key to achieving success in board exams, coupled with a strong work ethic, discipline, and adaptability. During my BSN course, I faced numerous challenges, such as balancing clinical rotations with study time. By pushing through these obstacles and maintaining my focus on long-term goals, I managed to stay on track and perform well in the exams." (Alumni 2022c). "The ability to bounce back from setbacks and stay focused was crucial. I remember struggling with a particularly tough subject, but through perseverance and additional study sessions, I was able to improve my understanding and perform well in the board exams." (Alumni 2018b)	"Resilience and determination are emphasized through challenging assignments and supportive guidance. For example, we often assign rigorous case studies that push students to their limits, preparing them for both exams and professional life. This approach not only enhances their problem-solving skills but also fosters the perseverance needed for success."(Faculty7). "We ensure that our students develop resilience by incorporating feedback loops and real-world challenges in their coursework. This method helps students learn to manage setbacks effectively and builds their determination to succeed." (Faculty2)
		Ethical Judgment and Integrity	"Maintaining honesty and integrity is crucial for a successful and ethical nursing career. I remember an instance where I had to choose between taking an easy route in my assignment or doing the work honestly. I opted for the latter, and it reinforced my commitment to ethical practice, which I believe was instrumental in my success in the board exams." (Alumni 2023d). "Honesty in academic work helped me build a strong foundation of trust and respect with my faculty and peers. This was crucial when preparing for board exams, as it fostered a supportive environment where ethical practice was a priority." (Alumni 2022b)	"Instilling ethics and integrity is essential for students to navigate their academic and professional careers with moral clarity. We emphasize these values through interactive discussions and ethical dilemma scenarios, ensuring students understand their importance in real-world situations." (Faculty5). "Incorporating ethics into the curriculum helps students understand their responsibilities and make sound decisions. We use case studies and role-playing exercises to illustrate the importance of integrity in nursing practice." (Faculty4)
	Faculty Influence and Support	Role Modelling and Professional Behaviour	"Observing faculty who exhibit professional behaviour and critical thinking inspires students. For example, one of my instructors consistently demonstrated thoughtful decision-making in clinical settings, which guided me in developing similar critical thinking skills crucial for board exams and my nursing career." (Alumni 2022a). "Faculty members who showed dedication and professionalism in their teaching and clinical supervision set a high standard for us. Their behaviour taught me the importance of being meticulous and thoughtful in my own practice." (Alumni 2023c)	"Role modelling prudent decision-making is key in influencing students to critically evaluate situations. We often use case studies and real-life scenarios to demonstrate how to handle complex situations, helping students apply theoretical knowledge to practical problems." (Faculty9). "We model professional behaviour by maintaining high standards in our interactions with students and by providing them with opportunities to observe and practice professional skills in realistic settings." (Faculty10)
		Empathy and Student Support	"Support and encouragement from empathetic faculty greatly impacted my confidence and preparedness for board exams. I recall a specific instance where a faculty member took extra time to address my concerns about a difficult topic, which significantly boosted my confidence and understanding." (Alumni 2023a). "The genuine care and understanding from faculty, such as offering emotional support during stressful periods, helped me stay motivated and focused. Their willingness to listen and provide personalized advice was invaluable." (Alumni 2018a)	"Being approachable and empathetic allows students to feel comfortable discussing concerns. For example, we hold open office hours and provide additional support sessions, which align with our core value of caring for others and help students navigate their academic challenges." (Faculty2, Faculty7). "Empathy in teaching is demonstrated through our efforts to address students' individual needs and provide a supportive learning environment. This approach helps students feel valued and supported throughout their academic journey." (Faculty3)
		Structured Guidance and Feedback	"Structured guidance and feedback from faculty were crucial in improving my performance, essential for board exam success. Regular feedback sessions helped me identify my strengths and areas needing improvement, which guided my study efforts effectively." (Alumni 2023e). "Detailed feedback on assignments and practice exams was invaluable. It allowed me to focus on specific areas that needed improvement and gave me a clearer understanding of how to approach the board exams." (Alumni 2022d)	"Creating an inclusive environment and offering constructive feedback helps students identify strengths and areas for improvement. Our feedback sessions are designed to provide students with clear guidance on how to enhance their performance and prepare for exams." (Faculty11, Faculty3). "We provide structured feedback through detailed evaluations of students' work, which helps them understand their progress and areas for growth. This systematic approach is crucial for preparing students for their board exams." (Faculty6)
	Curriculum and Character Development	Outcome-Based Education and Self-Reliance	"Outcome-based education taught me responsibility for my learning. I remember a project where I had to independently research a topic and present my findings. This experience not only prepared me for board exams but also instilled a sense of self-reliance and accountability." (Alumni 2023b). "The emphasis on meeting specific learning outcomes in our coursework pushed me to take charge of my own education. This self-directed approach was instrumental in my preparation for the board exams." (Alumni 2023f)	"Outcome-based education fosters self-reliance by focusing on students' ability to meet specific learning outcomes. Our curriculum is designed to encourage students to take ownership of their learning, which contributes to their success in board exams." (Faculty1). "By aligning our teaching methods with outcome-based education principles, we help students develop the skills needed for independent learning and self-motivation, which are essential for board exam success." (Faculty5)
		Holistic Curriculum Design	"The curriculum, which emphasized empathy and ethics, prepared me for both board exams and nursing practice. For instance, modules that incorporated case studies on ethical dilemmas helped me understand and apply these concepts effectively during my exams." (Alumni 2018b). "The holistic approach of our curriculum, which includes not only academic content but also character development, was crucial. It helped me build the necessary skills and values for both the board exams and my future nursing career." (Alumni 2019b)	"Integrating ethics and empathy-building activities throughout the curriculum enhances students' development. We incorporate ethical scenarios and empathy exercises into various courses, which prepares students for both their exams and professional practice." (Faculty9, Faculty6). "Our curriculum is designed to address both academic and personal growth. We include elements such as teamwork and ethical decision-making to ensure students are well-rounded and prepared for the challenges of the nursing profession." (Faculty5)
		Innovative Teaching Practices	"Innovative teaching methods, such as interactive simulations and Philips 66 with Case Base Learning, made learning more engaging and effective. These methods not only improved my understanding of complex concepts but also made studying for the board exams more enjoyable." (Alumni 2022d). "The use of technology and interactive tools in our classes made a significant difference. For example, virtual simulations of clinical scenarios provided practical experience that was invaluable for board exam preparation." (Alumni 2022g)	"Creative and innovative teaching strategies sustain students' focus and contribute to their development. We incorporate various teaching tools and techniques to keep students engaged and enhance their learning experience, which ultimately supports their success in exams." (Faculty5, Faculty3). "We use innovative methods, such as simulations and case-based learning, to create dynamic and effective learning environments. These strategies not only improve students' understanding but also prepare them for real-world challenges." (Faculty8)
	Personal and Professional Growth	Personal Growth and Self-Development	"Personal qualities like grit and self-discipline were crucial for my success. I faced challenges during my studies, but maintaining a strong sense of responsibility and confidence helped me overcome obstacles and excel in the board exams." (Alumni 2018a). "Developing self-confidence through personal growth activities was essential. I engaged in various reflective practices, especially during our clinical exposures that helped me build resilience and a positive mindset, which were crucial for my success in the board exams." (Alumni 2023d)	"Encouraging personal growth activities, such as reflective practices daily after their clinical rotations and goal-setting, helps students develop the qualities needed for success. We integrate these practices into our programs to support students' overall development and exam performance." (Faculty2). "We focus on fostering personal growth by providing opportunities for self-reflection and development. These activities are designed to build students' confidence and self-efficacy, which are vital for both academic and professional success." (Faculty11)
		Learning Environment and Support Systems	"The learning environment, including exploration and holistic development, played a significant role in my success. The opportunity to engage in diverse learning experiences and receive individualized feedback greatly enhanced my preparation for the board exams." (Alumni 2023e). "A positive and supportive learning environment, where faculty were open to feedback and provided resources, helped me stay motivated and excel in my studies. This environment was crucial during my preparation for the board exams as well." (Alumni 2022a)	"A supportive and inclusive learning environment, along with strong peer support networks, significantly enhances students' performance. We create a collaborative atmosphere that encourages mutual support and shared learning, contributing to students' success." (Faculty6). "We ensure that our learning environment is supportive and inclusive by providing ample resources and fostering a culture of collaboration. This approach helps students feel engaged and supported, which contributes to their academic success." (Faculty4)
		Peer Support and Collaboration	"Peer support and collaboration were vital for my academic success. Working with peers who shared similar goals and challenges provided motivation and fostered a sense of community that was instrumental during my board exam preparation." (Alumni 2019a). "Collaboration with classmates during study sessions was incredibly helpful throughout the years of my academic journey. We exchanged insights and strategies, which not only improved my understanding of the material but also made the preparation for board exams more effective." (Alumni 2023a)	"Fostering a collaborative learning atmosphere and encouraging peer support contribute greatly to students' success. We emphasize teamwork and peer interactions in our curriculum, which helps students support each other and excel in their exams." (Faculty2). "Encouraging peer interactions and collaborative learning helps students develop critical teamwork skills. This approach not only supports their academic performance but also prepares them for collaborative roles in their professional careers." (Faculty7)

Curriculum and character development and Personal and professional growth further explore the impact of education on student success. The curriculum, through outcome-based education and innovative teaching practices, promotes holistic development, equipping students with both academic and ethical competencies. Additionally, a supportive learning environment that encourages personal growth and peer collaboration is essential for both academic achievement and readiness for professional practice. Together, these themes underscore the critical role of character development in fostering success in nursing education.

## Discussion

This study underscores the pivotal role that character traits play in the consistent success of nursing graduates in the NLE in the Philippines, as evidenced by a 100% passing rate over the last 10 consecutive nationwide examinations. The results show that, in addition to academic proficiency, alumni and faculty character strengths play a significant role in producing this amazing result [15].

According to the demographic insights, a considerable proportion of the participants are recent graduates, with a preponderance of female graduates that is consistent with current nursing practice trends [16]. Given that most of the participants were from 2022 and 2023, the findings are especially relevant to modern nursing education and the challenges that newly graduated nurses face in the workplace. The younger alumni, most of whom are as young as 21 and 23, stress how crucial it is to understand how personality traits impact candidates' preparedness for board exams and clinical practice [12].

The VIA Institute of Character Strengths, which offers a scientifically verified assessment instrument to determine an individual's profile of character strengths, was central to the study design. This validated tool consists of 96 questions and measures 24 character strengths [14]. It provided a reliable basis for quantifying the character traits that contribute to success in both academic and professional settings [17]. The study’s use of this tool ensured that the character strengths measured were aligned with those identified as crucial for success, such as resilience, empathy, and ethical judgment. For instance, the high factor loadings for traits such as resilience and empathy in the data analysis confirm their significant role in academic and professional success.

The study explains and defines character qualities associated with success on the board exam; nonetheless, it does not prove a direct link between these attributes and exam results. However, the alignment of the VIA Inventory's measured traits with the study’s findings supports the conclusion that these character traits are crucial for managing the pressures of board exams and professional challenges. Qualities such as humility, resilience, and ethical judgment help deal with the pressures of board exams and the challenges that come with being a professional [17]. Resilience, in particular, guides students on how to manage the pressures of studying for exams and meeting clinical responsibilities [18]. The interplay between these character traits and academic success is further strengthened by qualitative insights gathered from interviews with both nursing alumni and faculty members. These characteristics not only promoted peer collaboration and a sense of accountability but also created an atmosphere where students helped one another along their educational paths [16].

The influence of teachers on the advancement of learners is one of the study's main areas of focus. Faculty members' use of the VIA Inventory to emphasize traits such as kindness, honesty, and empathy aligns with the tool’s validation and the study's findings. Their mentoring approaches and personal qualities can significantly influence how successfully students prepare for board examinations [11]. The alignment of faculty values with the character traits they instill in their students creates a supportive learning environment conducive to achieving a 100% passing rate [19]. The qualitative data reveals that effective mentorship and faculty support significantly influence students’ confidence and preparedness for board examinations [18]. In addition to offering structured criticism and creating a culture of ethical practice and ongoing learning, faculty members also act as role models [11]. An increasing recognition of the emotional and ethical complexity of nursing is demonstrated by faculty members' increasing emphasis on traits of spirituality and love as they mature in their careers [20]. Incorporating character education further reinforces a holistic framework into the curricula [15]. By encouraging students to cultivate empathy, resilience, and ethical decision-making alongside their academic skills, character development can be embedded within the nursing program [11].

The call for innovative instructional practices can further underpin a character-focused approach by prioritizing active engagement and collaborative learning, which can lead to improved student outcomes and substantial preparedness for board examinations [11]. Moreover, findings suggest that a character-driven educational approach can have profound implications for nursing practice beyond board examination results. Handling ethically and emotionally challenging environments, nursing professionals known for traits such as empathy, resilience, and integrity thrive in their careers [19]. This character trait enhances the provision of quality patient care and the overall well-being of healthcare environments, which aligns with the scope of nursing practice. That is why the prioritization of character development in academic institutions, together with knowledge-driven skills, cultivates compassionate leaders in various fields [21]. The profound influence of character traits on NLE success, combined with academic excellence, was further proven by the findings of the study. The alignment of character development with several strategies in education is pivotal for registered nurses to excel in the future through extensive preparation and a supportive environment [22]. Adapting both the interplay of character and academic performance into nursing education, this study promotes having well-rounded nurses who are compassionate and equipped to face the challenges and demands of the healthcare field alongside excellent board exam passing results. Future research and innovations in the curricula and the importance of character development as a core element in nursing education guarantee that graduates are ready to conquer the real world by delivering quality patient care and facing a challenging workplace [23]. Additionally, while this study provides significant insights into the role of character traits, it is essential to acknowledge that other factors, such as the quality of instruction [24], socioeconomic background [25], and institutional resources [26], also contribute to educational outcomes. Future research should delve into these aspects to offer a more comprehensive view of their influence in conjunction with character traits. Furthermore, engaging with conflicting studies and exploring theoretical advancements based on these findings could enhance academic value and provide a broader understanding of the dynamics at play.

Limitations and future research directions

Exploration of the potential impact of socioeconomic background and institutional support was not employed in the study, and the cultural factors that could influence the experiences and outcomes in Philippine education were not addressed. In addition, the study was conducted on one academic institution and used a small sample size that limited the generalizability of the findings. These limitations need to be subjected to further studies to gain a comprehensive understanding of the factors influencing board exam success and to support the development of more effective educational strategies considering diverse samples, a range of variables, and other contextual factors.

Recommendations

The study shows that character attributes, in addition to the intellectual capacity of a student, have an impact on future success. Traits such as empathy, resilience, and ethical judgment further influence passing the NLE [11]. This proves how moral instruction, in addition to intellectual content, has an impact on future success. For character education programs to be successful, nursing programs are suggested to incorporate service-learning projects [27], reflective writing [28], and role-playing exercises [29]. Moreover, the establishment of continuous programs for faculty development and structured mentorship programs can help teachers and students demonstrate the core values of wisdom, compassion, and mindfulness [30].

Peer support and trust between students and faculty are essential in creating a supportive learning environment [16,17], and measures to help manage the pressures of nursing education, such as student support services, resilience training, and counselling, need to be implemented [22]. The alignment of the core values to institutional policies will promote academic success and character development balance. To continually refine strategies in education and prepare graduates for the healthcare profession, the impact of character traits on nursing outcomes should be further investigated in future research.

## Conclusions

The study demonstrates that character traits are essential to the consistent 100% passing rate of nursing graduates in the NLE. While academic knowledge is indispensable, traits such as empathy, resilience, and moral judgment, along with honesty, teamwork, responsibility, and adaptability, have been found to significantly influence success. These findings highlight the importance of aligning nursing education with the university’s core values of inner goodness, excellence, and genuine concern for others. To ensure that character development is not only a concept but also a practical component of nursing education, institutions must foster a learning environment that integrates these traits into daily academic and clinical practice through mentorship, peer collaboration, and ethical decision-making. By embedding character education deeply into the curriculum, nursing programs can produce graduates who are not only competent in their field but also compassionate and ethical leaders in healthcare.
